# Effect of an eLearning Intervention on Undergraduate Health Professional Student’s General Histology and Embryology Summative Examination Scores

**DOI:** 10.4236/oalib.1106230

**Published:** 2020-06-18

**Authors:** Ian G. Munabi, Erisa S. Mwaka, Gonzaga G. Kirum, Haruna Kirwowa, Aloysius Gonzaga Mubuuke, Sarah Kiguli, William Buwembo

**Affiliations:** 1 Department of Anatomy, School of Biomedical Sciences, Makerere University College of Health Sciences, Kampala, Uganda; 2 Department of Radiology, School of Medicine, Makerere University College of Health Sciences, Kampala, Uganda; 3 Department of Paediatrics, School of Medicine, Makerere University College of Health Sciences, Kampala, Uganda

**Keywords:** eLearning, Interaction Equivalency Theorem, Bayesian Multilevel Modelling

## Abstract

Sub-Saharan Africa has the worst global shortage of health professionals. The use of eLearning interventions, that lead to increased interactions according to the Interaction Equivalency theorem, is a potential means of addressing this shortage of health professionals. In this audit we set out to determine the effect of an eLearning general histology and general embryology intervention on student’s summative examination scores. The audit compared the written and practical summative examination scores of three sets of student examinations one of which had a five-week eLearning intervention. Two of the examinations were for the same students in one year but different courses while the other two were for students doing the same subject but in different years. In each of the above pair of examinations there was one group from the course that had the eLearning intervention. A Bayesian multilevel regression modelling approach was used to analyse the student scores. The course with the online eLearning intervention had significantly better scores (p-value < 0.01), than the course on a different subject offered at the same time to the same students without the intervention and the same course with students from the previous academic year. On controlling for other factors, the eLearning intervention led to higher examination scores though this was not significant. Student’s nationality, sponsorship and program significantly affected the examination scores, controlling for other factors. Overall the students in the course with the online eLearning intervention had significantly better examination scores. The student’s nationality, sponsorship and program significantly affect their examination scores. Future, larger and or qualitative studies, are needed to further explore the effect of these factors on student’s examination scores.

## 1. Introduction

As of 2013, the World Health Organization, estimated that sub-Saharan Africa had the worst global shortage of health professionals with only 0.3 physicians per 1,000 population compared with 3.2 physicians per 1,000 population in Europe and 1.4 per 1,000 worldwide [1]. Africa also grapples with sub-optimal learning infrastructure and limited un-equitable distribution of human resource despite increasing student numbers in health professions training institutions [2]. The use of eLearning has been identified as one of the innovative ways to overcome the lack of infrastructure and other resources needed to support robust health professional training in low resource settings to address the shortage of health professionals [3] [4] [5]. In addition eLearning modules can be delivered online, at any location, on an as-needed basis to multiple consumers, at a low cost [4]. Based on evidence from a meta-analysis, eLearning using mobile devices has been found to be as effective as traditional learning for health professions education [6].

It is widely accepted that learning takes place through active engagement rather than passive transmission [7]. This active engagement, that is also described as interaction, forms the basis for the Interaction Equivalency Theorem, which emphasises that one of the three: student-teacher, student-student, and student-content interactions needs to be at a high level for deep learning to occur [7] [8] [9]. In this study, we proposed an eLearning educational intervention for learning general histology and general embryology among health professional students. The intervention aimed to enhance the current learning environment and create a more positive learning experience for students through increased student-content and student-student interactions in comparison to other courses as shown in Table 1. It is these increased student learning interactions that may in part explain the previously mentioned increased effectiveness of eLearning [6]. There is an increasing recognition for the need for evidence to support these interactions as a result of eLearning interventions for improved learning outcomes [10]. In this study, we set out to determine the effect of an eLearning intervention on students’ summative assessment scores in general histology and general embryology.

## 2. Methods

### Setting

This audit was done at Makerere University College of Health Sciences (MakCHS) Department of Anatomy, that currently admits an average of 350 undergraduate students annually into the following health professional programs: Bachelor of Medicine and Bachelor of Surgery, Bachelor of Nursing, Bachelor of Medical Radiology, Bachelor of Dentistry, Bachelor of Biomedical Sciences and Bachelor of Pharmacy [11]. The majority of the students on these programs join directly from secondary school as 19 to 20-year olds. As a mandatory first year course for all the above programs the class sex composition is the similar to the admission ratios with more male than female students [2]. Each of these programs requires that students learn different aspects of the cell and related basic general histology and general embryology during their first year of study.

### Process of previous training

During the first semester of the first-year, general histology and general embryology training at MakCHS Department of Anatomy involves the use of lectures, practical and selected readings from Wheatears functional general histology. The schedules for a typical week for the first 15/17 weeks of this first semester are shown in Table 2. The one-hour lectures, which cover different topics related to general histology and general embryology are taught by faculty. This is done through presentations on different aspects of general histology and general embryology of importance and their related clinical applications. The practical sessions are two hours of real-time microscopy work where students get to view different slides using the available 20-working microscopes. In each session, it is common to have up to eight students sharing one microscope. The teaching of general histology runs concurrently with that of the gross anatomy of the limbs for this semester with both the practical and teaching time split between the two subjects (see Table 2).

### The eLearning training Intervention

**Figure 1.**
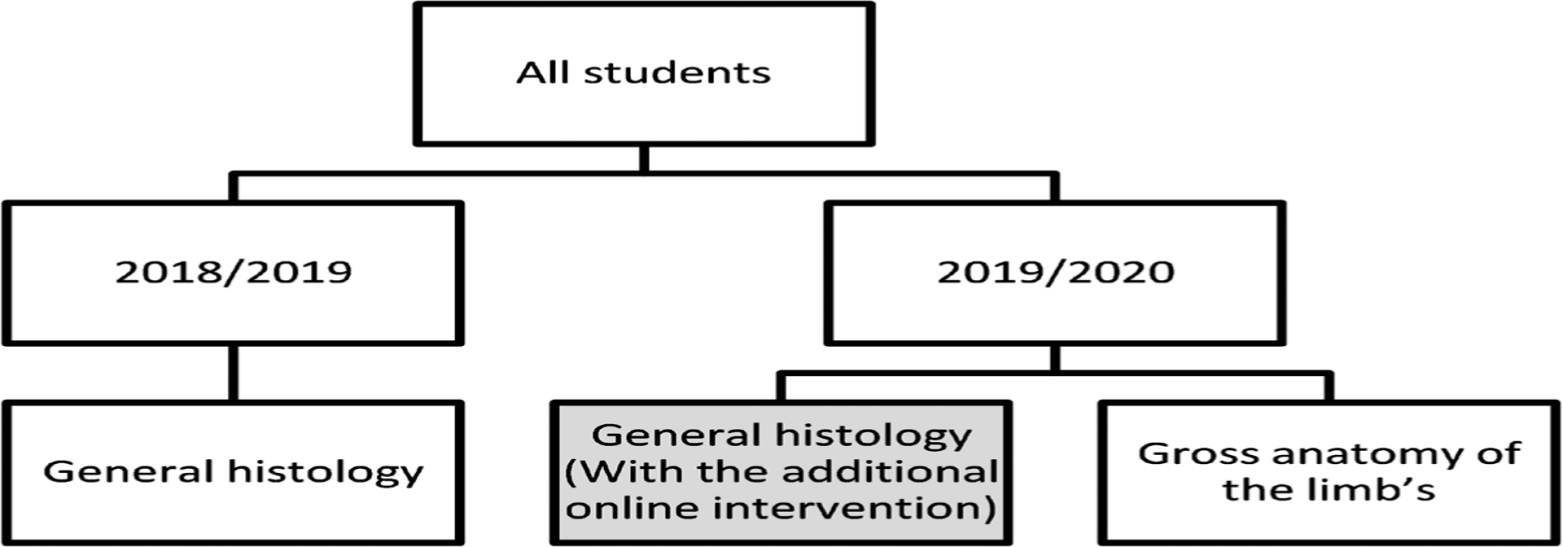
Study design.

Table 1 presents a summary of the similarities and differences in educational content, assessment activities and other descriptive information for the courses analysed in this study. In Table 1, note that it was only the students on the academic year 2019/2020 general histology and general embryology course that were exposed to the online eLearning intervention. The eLearning online intervention for the 2019/2020 student intake (see Figure 1), involved the design of a five-week, Moodle (https://muele.mak.ac.ug/), cells and tissues course, to cover the following aspects of the general histology and general embryology course: 1) weekly prescribed readings from the Wheatears functional general histology, 2) case scenarios featuring selected journal article readings showing applications of the identified knowledge, and (3) weekly unsupervised open book practice quizzes based on the given material that the students could attempt up to a maximum of three times. The quizzes were designed using the Moodle quiz module and used the multiple choice, extended matching, and labelling of microscope images. Because some of the students had challenges and missed participating in the online course, none of the online quiz results were included in the computation of the final summative examination scores. The summative examination had a two-hour written theory examination that was composed of 100 multiple choice questions and a previously described one minute per station steeple chase examination that had 10 stations dedicated to general histology and general embryology questions [11]. The content of the summative examinations for the two academic years: 2018/2019 and 2019/2020 remained the same in scope and content.

### Sample size and score selection

The target sample size of 879 students’ examination scores was calculated using the free online http://www.openepi.com online sample size calculator for sample sizes based on comparing two means for the following considerations: power = 0.8, alpha = 0.95, ratio of unexposed/exposed = 2:1, assumed mean difference = 2 and assumed standard deviations in the unexposed = 10.02 and exposed = 8.34. This gives a total of 266 student scores for each exam or 798 students’ examination scores in total. To this was included a 10% allowance for the design effect that brought the final sample size to 293 scores per group or 879 scores. The scores for the students in the academic year 2019/2020 for the general histology and general embryology who did the online course were matched with the scores from the same students for the summative examination on gross anatomy of the limbs. Only the scores of the students sitting any one of the selected examinations for the first time were included [11]. A random sample of scores was obtained using a computer-generated list of random numbers corresponding to the study identifier numbers for each set of examination scores.

### Analysis

Analysis of the three sets of summative student examination scores involved the generation of descriptive statistics using the independent samples t test for the 2018/2019 and 2019/2020 academic year histology and embryology exam comparisons and paired samples t test for paired comparisons between the scores for 2019/2020 academic year histology and gross anatomy of the limbs in the R statistical computing environment [12]. This was followed by a univariable then multivariable multilevel Bayesian gaussian regression analysis to determine the differences in scores for the three groups. The multilevel Bayesian gaussian regression analysis using the Brms package [13] [14], was selected in view of the small sample population, small mean differences and the lack of independence in the scores (unit of analysis) from the students (see Figure 1). The modelling used the following: number of chains = 4, cores = 2, seed = 12345, iterations =20,000, “adapt delta” = 0.99 and default for the other parameters for the univariable modelling and chains = 6 and cores = 3, for the multivariable modelling. For adequate mixing of the chains the modelling output had to have a Rhat value of 1.01 or less, and both the bulk (Bulk ESS) and tail (Tail ESS) effective sample sizes of more than 100 * Chains (400), for inclusion in the final analysis [14]. The other R statistical computing environment [12], packages used included: readerxl [15], tidyr [16], dplyr [17], psych [18], and summary tools [19]. The results were summarised in tables and figures. For all the analyses the cut off for significance was set at 0.05. Any record with missing information was deleted from the dataset prior to analysis.

### Ethical considerations

This was an audit using the examination results following an educational training intervention by the Anatomy Department. As an equivalent of the NIH category 2, exemption 2 type of study, the work presented in this manuscript did not require ethical review due to: 1) the associated low risk of harm to the participants; 2) use of deidentified educational achievement scores that had already gone through the assessment procedures that includes public display on notice boards; 3) the scores were the products of the assessment process run by the department for this course; and 4) no student names or other identifiers were used during analysis or in the manuscript.

## 3. Results

A total of 1,219 student examination scores were found in the examination records. There were 85 from students repeating one of the examinations and another 129 records with missing information that were deleted. A total of 293 randomly selected student’s scores were obtained from each of the 2018/2019 and 2019/2020 academic years general histology and general embryology examinations using computer generated numbers. The academic year 2019/2020 gross anatomy of the limb’s examination had 246 matching student examination scores with student identifiers corresponding to the already included 2019/2020 academic year general histology and general embryology examination that were included in the final dataset of 832 student examination scores.

Table 3 provides a summary of the descriptive information about the students and the test scores that were selected in the final sample of student’s examination scores. From this table note that the 2018/2019 general histology and general embryology examination score was lower than that of the 2019/2020 general histology and general embryology examination score when the intervention was provided. This difference was significant (Difference = −4.04, 95% CI: −6.02 to −2.06, p-value < 0.01). A similar observation was made for the two practical examination scores whose difference was significant (Difference = −5.54, 95% CI: −8.78 to −2.29, p-value < 0.01). The 2019/2020 general histology and general embryology examination scores, the intervention group, were significantly higher than the corresponding paired gross anatomy of the limb’s 2019/2020 examination student scores (Difference =14.09, 95% CI: 12.31 to 15.86, t = 15.63, df = 245, p-value < 0.01). A smaller but significant difference was observed in the paired practical examination scores of the same student for these two examinations (Difference = 3.96, 95% CI: 1.33 to 6.60, t = 2.96, df = 245, p-value < 0.01).

Table 4 summarises both the univariable and multivariable multilevel regression analysis for the different observations. In this table note that the shaded cells represent significant observations and that the values for the bulk (Bulk ESS) and tail (Tail ESS) effective sample sizes are all greater than the minimum number of 400. For the multivariable model, keeping other factors constant, students exposed to the online intervention had higher scores for both the written and practical examinations. This was not significant. In the written examination multivariable model, keeping other factors constant, on average, the local Ugandan students, had significantly higher scores than their international classmates (posterior mean difference = 9.49, 95% CI: 6.19 to 12.77). The privately sponsored students had significantly lower scores that the Government of Uganda sponsored students on both the written (posterior mean difference = −2.36, 95% CI: −3.82 to −0.92) and practical examinations (posterior mean difference = −3.78, 95% CI: −6.29 to −1.32). In this table also note that there were significant differences in scores of students on different programs when compared with the Biomedical Sciences program. The practical examination had a group (examination) level intercept was 10.12 (95% CI: 1.94 to 31.62) and sigma estimate of 15.68 (95% CI: 14.95 to 16.45). For the written examination the group (examination) level intercept was 10.39 (95% CI: 3.36 to 27.15) and sigma estimate of 9.23 (95% CI: 8.80 to 9.69). The intraclass correlation for the practical examination model was 0.29 (95% CI: 0.02 to 0.79) while that for the written examination was 0.56 (95% CI: 0.13 to 0.89). The fit for the practical examination model (R^2^ = 0.16, 95% CI: 0.12 to 0.20) was smaller than that for the written examination model (R^2^ = 0.38, 95% CI: 0.34 to 0.42).

## 4. Discussion

We set out to determine the effect of an eLearning training intervention on overall summative assessment scores of students in general histology and general embryology. Overall, we found that the group that had received the online educational intervention performed significantly better than their colleagues did on a similar examination administered in the previous year with no online intervention (p-value < 0.01) and themselves in a related subject (gross anatomy of the limb’s, p-value < 0.01) in the same semester. This pattern of better scores, in the 2019/2020 general histology and general embryology examination compared to the other examinations, was observed in both the written and practical aspects of the summative examination. Multiple Choice Questions (MCQs), which gave the written summative examination scores that were analysed, have both good reliability and validity. The way the MCQs and the practical examinations are used in this context makes them a good measure of knowledge/understanding and or recall abilities of the students. Based on this we conclude that overall the students in the intervention group attained a better coverage of the required knowledge for the subject.

It is also of interest to note that the same students scored better on the 2019/2020 general histology and general embryology examination compared to the gross anatomy of the limbs. This is shown by the observed significant difference in mean summative examination scores for the two examinations using the paired samples T test (p-value < 0.01). This was observed for both the written and practical examinations. This is best understood when viewed through the lens of the Interaction Equivalency Theorem [9] which as previously described, states that as long as one of the interactions between either: student-content, student-teacher and student-student is at a heightened level, there will be meaningful learning even when the other two are minimised [9] [20]. For these two courses involving the same set of students it is possible that the online eLearning course interventions requirements led to more student-content through the use of additional reading materials and progressive online tests that provided immediate feedback to support continued learning (see Table 1). The observed differences in scores for the same student, in the same semester, may thus be attributed to enhanced student-content and student-student interactions in the 2019/2020 general histology and general embryology examination as a result of the eLearning intervention.

On controlling for other factors using multilevel regression analysis (Table 3), the students who were exposed to the online eLearning intervention had on average 7.90% higher written examination scores for the 2019/2020 general histology and general embryology compared with both the 2018/2019 general histology and general embryology and 2019/2020 gross anatomy of the limbs scores. This was not significant. The range for the possible location of this difference remained relatively unchanged with both the univariable and multivariable models. A similar observation is made for the effect of the online eLearning intervention on the practical examination scores. Note again that as with the written examination the difference in scores due to the eLearning intervention remain unchanged for both the univariable and multivariable modelling. This is evidence for the relative independence of the effect of the eLearning intervention from the other factors in the model.

The other factors with significant differences on multivariable modelling for the written and practical examinations as shown in Table 3 were: 1) The nationality of the students, where the local Ugandan students performed significantly better than the international students. This being the first semester for the students, it is possible that the international students were still settling into their new environment and learning both the language and culture that may have affected their scores. This need to learn a new language for example may in part explain why nationality did not have a significant effect on the practical examination scores whose questions are presented as simple statements [11], compared to the higher level of comprehension required to answer the multiple-choice questions in the written examination. 2) Sponsorship status where the privately sponsored students scored significantly worse than the government sponsored students. The privately sponsored students, who may be working to earn their fees and at the same time study, are usually also admitted with lower scores than the students on the government merit-based scholarship scheme. This was significant for both the practical ad written examinations. 3) Program, where for the written the students on Medicine, Dentistry and Pharmacy programs on average performed significantly better and those on the Radiology programs on average performed significantly worse than the reference Biomedical Sciences program students. For the practical it was only the students on the Nursing and Pharmacy programs that had significantly worse examination scores. In view of the fact that all the students went through the same exposure with the exception of the intervention, these differences may be due to factors unique to each of the above groups of students.

One of the key limitations to this analysis was the presence of very small differences for the different observations affecting the scores of both the written and practical examinations. This is emphasised by the poor, for the practical examination (0.29) and moderate, for the written (0.56) examination, values of the intraclass correlation coefficients [21]. This limitation was partly overcome through the use of distribution free Bayesian approach to the modelling, that also allows for estimation of the observed effects using the assumption of sampling from a large population. This gave narrow confidence intervals for the possible location of the observed mean differences in examination scores due to the different observations in the models as summarised in Table 4. The other limitation is the possible effect of the reduction in the class size for the two academic years that the use of a random sample of scores for analysis may not have completely removed (see Table 1). Future studies may need to consider using many more clusters of scores to address this. Thus, there is a need for cautious extrapolation of the presented findings to other populations. We thus recommend the replication of this study in other settings for additional comparisons.

## 5. Conclusion

Overall the students in the course with the online eLearning intervention had significantly better assessment scores than the controls. The student’s nationality, sponsorship and program significantly affect their examination scores. In view of the observed small differences, there is a need for larger and or qualitative studies, to further explore the effect of these factors on student’s examination scores.

## Figures and Tables

**Table 1.** Summary of course related educational activities prior to each examination.

Criteria		Examination	
Academic year	2019/2020	2019/2020	2018/2019
Subject	General histology and embryology	Gross anatomy of the limb’s	General histology and embryology
Practical activity	Microscopy	Gross anatomy	Microscopy
Number of lecture hours	15.5	36.5	13.5
Number of scheduled practical hours	60	60	57
Core textbook	Wheatears	Cunningham vol. 1	Wheatears
Number of progressive tests	1	2	2
Number of students taking course	375	349	495
**Intervention**			
Online course exposure	Yes	No	No
Online additional Journal paper readings	25	0	0
Number of online practice tests	5	0	0

**Table 2.** Weekly semester one anatomy schedule.

Day	Time	Activity	Duration
Monday		*Other subjects*	
Tuesday	Morning	Lectures (1-hour-Gross and 1-hour-General histology)	2 hours
	Afternoon	Practical’s: Group A (General histology)/Group B (gross)	3 hours
Wednesday		*Other subjects*	
Thursday	Morning	Lectures (1-hour-Gross and 1-hour-General histology)	2 hours
	Afternoon	Practical’s: Group A (Gross)/Group B (General histology)	3 hours
Friday		*Other subjects*	
Weekend	All day	Self-directed learning and or Gross Anatomy	

**Table 3.** Descriptive information of the students and scores.

Observation	Freq (% Valid)
Sex	
Female	226 (27.16)
Male	606 (72.84)
Nationality	
International	35 (4.20)
Ugandan	797 (95.79)
Sponsorship	
Government of Uganda	411 (49.40)
Private sponsored	421 (50.60)
Online eLearning intervention	
Exposed	293 (35.22)
Controls	539 (64.78)
Test scores	
Histology	586 (70.43)
Gross anatomy of the limb’s	246 (29.57)
Program	
Biomedical Sciences	128 (15.38)
Cytotechnology	55 (6.61)
Dentistry	80 (9.62)
Medicine	356 (42.79)
Nursing	60 (7.21)
Optometry	25 (3.00)
Pharmacy	84 (10.10)
Radiology	24 (2.88)
Speech and Language	20 (2.40)
**Total**	**832 (100)**
Test scores	Mean (N, SD)
Histology and embryology 2019/2020 written	72.55 (293, 9.35)
Histology and embryology 2018/2019 written	68.51 (293, 8.44)
Gross anatomy of the limb’s 2019/2020 written	58.82 (246, 12.79)
Histology and embryology 2019/2020 practical	56.23 (293, 13.98)
Histology and embryology 2018/2019 practical	50.69 (293, 18.48)
Gross anatomy of the limb’s 2019/2020 practical	53.08 (246, 17.44)

**Table 4.** Summary of the regression analysis results.

Model	Univariable			Multivariable
Written examination				
Observations in model	Intercept (95% CI)	Value (95% CI)	Value	95% CI (Bulk ESS, Tail ESS)
Intercept	-	-	46.89	31.65 to 63.13 (10,955, 12,615)
Sex (Male)	66.15 (56.31 to 76.33)	1.23 (−0.32 to 2.77)	−0.50	−1.95 to 0.97 (28,356, 21,686)
Nationality (Ugandan)	56.51 (46.31 to 67.20)	11.07 (7.69 to 14.45)	9.49	6.19 to 12.77 (30,281, 22,415)
Sponsorship (Private sponsored)	69.64 (60.02 to 79.88)	−4.85 (−6.24 to −3.49)	−2.36	−3.82 to −0.92 (23,279, 21,819)
Program				
Intercept	64.45 (54.28 to 75.01)	-	-	-
Biomedical science	-	Ref.	Ref.	-
Cytotechnology	-	−0.79 (−3.96 to 2.38)	−0.88	−3.81 to 2.08 (18,244, 21,632)
Dentistry	-	4.31 (1.51 to 7.10)	3.71	1.08 to 6.30 (16,146, 20,328)
Medicine	-	5.00 (2.99 to 7.02)	2.80	0.79 to 4.80 (12,131, 18,405)
Nursing	-	−0.45 (−3.50 to 2.61)	−0.00	−2.94 to 2.94 (16,843, 20,051)
Optometry	-	1.17 (−3.13 to 5.44)	−0.62	−4.68 to 3.50 (22,291, 20,764)
Pharmacy	-	3.06 (0.33 to 5.85)	2.74	0.14 to 5.35 (16,591, 20,308)
Radiology	-	−4.03 (−8.45 to 0.36)	−4.26	−8.40 to −0.13 (23,474, 21,492)
Speech and language	-	−1.81 (−6.55 to 2.90)	−4.32	−8.75 to 0.16 (22,131, 22,298)
Online intervention (exposed)	64.10 (49.73 to 79.21)	9.03 (−20.19 to 38.93)	7.87	−23.07 to 38.38 (11,208, 10,782)
Practical exam results	56.99 (46.97 to 67.36)	0.19 (0.15 to 0.23)	0.16	0.12 to 0.20 (31,507, 22,494)
Practical examination				
Intercept	-	-	29.90	11.33 to 48.68 (13,302, 13,493)
Sex (Male)	52.15 (44.22 to 60.08)	1.74 (−0.83 to 4.30)	0.05	−2.46 to 2.56 (34,196, 20,602)
Nationality (Ugandan)	49.80 (40.33 to 59.39)	3.76 (−1.94 to 9.54)	−3.92	−9.51 to 1.72 (33,167, 23,406)
Sponsorship (Private sponsored)	56.47 (48.93 to 64.47)	−6.03 (−8.26 to −3.81)	−3.78	−6.29 to −1.32 (27,351, 22,714)
Program				
Intercept	52.53 (44.52 to 60.73)	-	-	-
Biomedical science	-	Ref.	Ref.	-
Cytotechnology	-	−1.76 (−6.98 to 3.57)	−1.87	−6.85 to 3.09 (21,943, 22,274)
Dentistry	-	0.87 (−3.72 to 5.45)	−2.21	−6.59 to 2.23 (19,182, 21,834)
Medicine	-	4.03 (0.73 to 7.36)	−0.25	−3.67 to 3.24 (14,711, 19,997)
Nursing	-	−8.39 (−13.39 to −3.35)	−9.46	−14.41 to −4.52 (20,691, 22,426)
Optometry	-	0.99 (−6.16 to 8.08)	−1.33	−8.17 to 5.54 (24,542, 22,514)
Pharmacy	-	−2.29 (−6.88 to 2.33)	−4.63	−9.07 to −0.23 (19,817, 21,710)
Radiology	-	−2.22 (−9.59 to 5.11)	−0.38	−7.45 to 6.65 (26,383, 20,615)
Speech and language	-	1.39 (−6.21 to 9.07)	−0.22	−7.88 to 7.40 (23,552, 21,761)
Online intervention (exposed)	51.94 (39.00 to 65.10)	4.46 (−19.53 to 29.47)	0.41	−32.18 to 33.20 (11,887, 10,412)
Written examination results	19.77 (8.15 to 30.70)	0.50 (0.40 to 0.61)	0.46	0.35 to 0.57 (32,876, 23,122)
